# Bioleaching of Indonesian Galena Concentrate With an Iron- and Sulfur-Oxidizing Mixotrophic Bacterium at Room Temperature

**DOI:** 10.3389/fmicb.2020.557548

**Published:** 2020-10-08

**Authors:** Siti Khodijah Chaerun, Edina Amadea Putri, Mohammad Zaki Mubarok

**Affiliations:** ^1^Department of Metallurgical Engineering, Faculty of Mining and Petroleum Engineering, Institut Teknologi Bandung, Bandung, Indonesia; ^2^Geomicrobiology-Biomining and Biocorrosion Laboratory, Microbial Culture Collection Laboratory, Biosciences and Biotechnology Research Center (BBRC), Institut Teknologi Bandung, Bandung, Indonesia

**Keywords:** semi-direct bioleaching, direct bioleaching, galena, lead (Pb), an iron- and sulfur-oxidizing mixotrophic bacterium

## Abstract

Biohydrometallurgy is believed to be a promising future study field for the recovery of lead (Pb) from ores/concentrates since the pyrometallurgical/hydrometallurgical processes have been largely applied to recover Pb to date, which operates at high temperature and generates volatile Pb matters that are hazardous and carcinogenic to human health. Hence, the main purpose of this study was to investigate the biohydrometallurgical extraction of Pb from the Indonesian galena concentrate through bioleaching using an iron- and sulfur-oxidizing mixotrophic bacterium (identified as *Citrobacter* sp.). The bioleaching experiments were conducted in shake flasks containing the modified LB broth medium supplemented with galena concentrate with a particle size of *d*_80_ = 75 μm at room temperature. Both semi-direct and direct bioleaching methods were employed in this study. The bacterium was able to extract lead (Pb) from galena concentrate with high selectivity to Cu and Zn (0.99 and 0.86, respectively). The highest extraction level of 90 g lead dissolved/kg galena concentrate was achieved using direct bioleaching method at bioleaching conditions of 2% *w/v* pulp density, 5 g/L FeCl_3_, 50 g/L NaCl, 20 g/L molasses and a rotation speed of 180 rpm at room temperature (25°C). The addition of FeCl_3_, NaCl, and molasses increased the lead leaching efficiencies, which were also evidenced by the FTIR, XRD, and SEM-EDS analyses. From industrial and commercial standpoints, the selective bioleaching represented in this study may be beneficial to the development of lead leaching from sulfide minerals, since insoluble anglesite (PbSO_4_) precipitates are formed during ferric sulfate oxidation, thus making the recovery of lead through bioleaching unpractical.

## Introduction

Recently, the extraction of lead (Pb) from sulfide ores relies on pyrometallurgical smelting processes and combined pyro/hydrometallurgical methods that are the high energy consumption and cause severe environmental pollution problems due to the emission of both SO_2_ and volatile lead matters. Hydrometallurgical routes of leaching lead concentrates have been developed progressively by testing a number of solvents to overcome such problems (Greet and Smart, 2002; Aydoğan et al., 2007; Long et al., 2009; Wu et al., 2014). However, the chemical solvents used are reported to have a very low solubility of lead, a high corrosiveness, and very high toxicity, and a significantly high temperature (65–85°C) is required to achieve a high lead recovery as listed in Table 1 (Warren et al., 1987; Pashkov et al., 2002; Aydoğan et al., 2007; Qin et al., 2009; Zárate-Gutiérrez et al., 2010, 2015; Baba and Adekola, 2013; Wu et al., 2014; Anugrah et al., 2017; Allen and Igboayaka, 2019).

**TABLE 1:** Previous studies on leaching lead (Pb) from pure galena, galena concentrates, and high-grade Pb-Zn bearing ores.

Ore/mineral	Leaching agent/lixiviant	Pulp density	Leaching efficiency	Leaching time, Temp, pH	Authors
Galena concentrate: PbS	0.2 M FeCl_3_ 2 M NaCl 0.1 M HCl	1% w/v pulp density	∼100% Pb	14 min (52°C, pH 2)	Warren et al., 1987
Galena concentrate: Pb (59.2 wt.%) Zn (2.6 wt.%)	1 M HNO_3_ 0.01 M Fe(NO_3_)_3_	5% w/v pulp density	90% Pb	60 min (50°C)	Pashkov et al., 2002
Galena concentrate: Pb (79.0 wt.%) Zn (1.90 wt.%) Cu (0.50% wt.%)	3 M CH_3_COOH 0.5 M H_2_O_2_	2% w/v pulp density	∼95% Pb	90 min (50°C, pH 5.1)	Aydoğan et al., 2007
Galena concentrate: PbS (77.24 wt.%) ZnS (0.65 wt.%)	75 g/L FeCl_3_.6H_2_O NaCl 250 g/L 0.1 M HCl	N/A	97.39% PbS converted to PbCl_2_	40 min (90°C, pH 2)	Qin et al., 2009
Galena concentrate: PbS (29.5 wt.%) ZnS (21.6 wt.%) CuFeS_2_ (1.4 wt.%)	0.65 M HNO_3_	10% w/v pulp density	80% Pb 100% Ag	90 min (130°C, pH 0.69)	Zárate-Gutiérrez et al., 2010
Galena ore: Pb (58.66 wt.%) Zn (0.16 wt.%)	1 M tributylphosphate in 100% MIBK	2% w/v pulp density	92.1% Pb	30 min (25°C, pH 5)	Baba and Adekola, 2013
Galena concentrate: Pb (54.27 wt.%) Zn (15.29 wt.%)	Acidic ferric methanesulfonate solution	0.4% w/v pulp density	∼100% Pb	10 min (65–85°C, pH < 3)	Wu et al., 2014
Galena concentrate: PbS (24.9 wt.%) ZnS (21.6 wt.%) CuFeS_2_ (1.4 wt.%)	1 M Na_3_Cit 0.04 M H_2_O_2_	10% w/v pulp density	100% Pb	120 min (25°C, pH 7)	Zárate-Gutiérrez et al., 2015
Galena concentrate: Pb (66.6 wt.%) Zn (7.38 wt.%)	3.44 M H_2_SiF 9.80 M H_2_O_2_	12% w/v pulp density	99.26% Pb	135 min (97°C)	Anugrah et al., 2017
High-grade Pb ore: Pb (31.75 wt.%) Zn (0.93 wt.%) Cu (0.53 wt.%)	2.15 M HNO_3_	20% w/v pulp density	∼33% Pb	150 min (70°C)	Allen and Igboayaka, 2019

Therefore, bioleaching of lead concentrates as a biohydrometallurgical method has emerged as a possible solution for overcoming the aforementioned problems since the bioleaching is a low-cost, environmentally friendly method. Of the bacterial genera and species, *Acidithiobacillus ferrooxidans* and *Acidithiobacillus thiooxidans* (chemolithotrophs) have been employed frequently for bioleaching sulfide ores (Garcia Jr. et al., 1995; Da Silva et al., 2003; Da Silva, 2004). However, the bioleaching of galena (PbS) has been poorly studied, since the complete oxidation of galena leads to insoluble anglesite (PbSO_4_) that thus precludes the recovery of lead from bioleaching and ferric sulfate leaching through conventional solvent extraction/electrowinning routes (Da Silva et al., 2003; Da Silva, 2004). Moreover, several studies demonstrated that the elevated lead bioleaching/leaching efficiencies were achieved in the presence of NaCl (Warren et al., 1987; Liao and Deng, 2004; Ye et al., 2017), FeCl_3_ (Dutrizac, 1986; Kim et al., 1986; Warren et al., 1987; Dutrizac and Chen, 1990; Long et al., 2009), and ferric (Fe^3+^) ions (Pashkov et al., 2002). Hence, the present study investigated the use of a local mixotrophic bacterium (identified as *Citrobacter* sp.) capable of oxidizing iron and sulfur as well as producing biosurfactants, including EPS (extracellular polymeric substances) in extracting lead from galena concentrate to enhance lead recovery. Compared to previous studies on galena (PbS) bioleaching and high-grade Pb ores bioleaching summarized in Table 2 (Bang et al., 1995; Garcia Jr. et al., 1995; Da Silva et al., 2003; Da Silva, 2004; Pacholewska, 2004; Jiang et al., 2008; Baba et al., 2011; Mejía et al., 2012; Ghassa et al., 2014), the present study is different from previous studies and more beneficial from metallurgical standpoints as follows: (1) the bacterium *Citrobacter* sp. employed in this study belongs to mixotrophic group capable of utilizing both organic and inorganic compounds for energy and carbon sources, thus making much easier for industrial applications since bacterial carbon sources can be derived from any organic wastes abundant in Indonesia and many other tropical countries; (2) the lead bioleaching in this study only employs a pure bacterial culture (herein *Citrobacter* sp.) that is able to oxidize both iron and sulfur as well as proliferate at high NaCl concentration and highly resistant to toxic metals, while previous studies mostly utilize a mixed culture of chemolithoautotrophs dominated by *Acidithiobacillus ferrooxidans*, *Acidithiobacillus thiooxidans* and *Leptospirillum ferrooxidans* that are very sensitive to organic compounds; (3) The lead bioleaching in this study takes place at room temperature (25°C) and the pH range of 3.5–4.6 that are more advantageous to energy-saving lead leaching process and the reduced usage of expensive anti-corrosive materials; (4) Medium used in this study contains NaCl, which elevates lead bioleaching efficiencies where NaCl can be replaced with seawater that is commonly used in many metallurgy and mining industries, thus providing a cost-effective lead leaching technology; (5) By using this local mixotrophic bacterium *Citrobacter* sp., the lead bioleaching from the galena concentrate is selective to Cu and Zn, thereby providing a selective lead leaching process that is profitable for subsequent Cu and Zn leaching processes since Pb (PbS) in association with CuFeS_2_ and ZnS always makes passivation layers that hinder Cu and Zn dissolution; (6) The bacterium *Citrobacter* sp. also produces biosurfactants (including EPS) that are useful for maintaining Fe^3+^ solubilization at pH > 4.0.

**TABLE 2:** Previous studies and the current study on bioleaching lead (Pb) from pure galena, galena concentrates, and high-grade Pb-Zn bearing ores.

Ore/mineral	Medium and pulp density	Microbes employed	Bioleaching efficiency	Bioleaching time, Temp, pH	Authors
Pure galena (PbS)	Mineral salts solution containing 0.4 g/L each (NH_4_)_2_SO_4_, MgSO_4_.7H_2_O, and K_2_HPO_4_ (2.5–5% w/v pulp density)	*Acidithiobacillus thiooxidans* or *Acidithiobacillus ferrooxidans*	1.1–5 mg/L Pb	29 days (24°C, pH 2)	Garcia Jr. et al., 1995
Pure galena (PbS): 85.6 wt.% Pb	MS medium containing 3 g/L (NH_4_)_2_SO_4_, 0.5 g/L KH_2_PO_4_, 0.5 g/L MgSO_4_ (2% w/v pulp density)	*At. ferrooxidans*	∼90% Pb	4 days (30°C, pH 2.8)	Bang et al., 1995
A natural galena: PbS (60.9 wt.% Pb) ZnS (15.2 wt.% Zn)	Modified Kelly Medium = MKM containing 0.4 g/L (NH_4_)_2_SO_4_, 0.4 g/L MgSO_4_.7H_2_O, 0.04 g/L potassium orthophosphate (10% w/v pulp density)	A mixed culture of *At. thiooxidans, At.ferrooxidans, Leptospirillum ferrooxidans*	34% Pb 1% Zn	6 days (35°C, pH 2)	Da Silva et al., 2003
A natural galena: PbS (60.9 wt.% Pb) ZnS (15.2 wt.% Zn)	Modified Kelly Medium = MKM (10% w/v pulp density)	A mixed culture of *At. thiooxidans, At. ferrooxidans, L. ferrooxidans*	34% Pb	6 days (35°C, pH 2)	Da Silva, 2004
Galena concentrate: PbS (∼80%) ZnS (∼5%)	2K medium (5% w/v pulp density)	*At. ferrooxidans*	43% Pb (9.8 mg/L Pb) 99% Zn (196 mg/L Zn)	16 days (25°C, pH 2)	Pacholewska, 2004
Galena: Pb (79.5 wt.%) Zn (1.92 wt.%) Cu (0.17 wt.%)	9K medium (3.8% w/v pulp density)	*At. ferrooxidans*	0.01098 mol/L (=2.27 g/L) Pb Zn (N/A)	6 days (30°C, pH 2)	Jiang et al., 2008
Galena ore: PbS (58.7%) ZnS (0.16%)	Agarose-simulated 9K medium (10% w/v pulp density)	A mixed culture of acidophilic bacteria predominantly *At. ferrooxidans*	89% Pb 92% Zn	5 days (35°C, pH 2)	Baba et al., 2011
Galena concentrate: PbS (90%) ZnS (7.5%) CuFeS2 (0.7%)	T&K medium (10% w/v pulp density)	A mixed culture of *At. ferrooxidans* and *At. thiooxidans*	57% Pb Zn (N/A)	30 days (30°C, pH 1.8)	Mejía et al., 2012
High-grade Zn–Pb bearing ore: PbS (12.4%) ZnS (40.71%)	9K medium (5% w/v pulp density)	A mixed culture of iron- and sulfur-oxidizing moderately thermophilic acidophilic chemolithotrophic bacteria	0.027% Pb 98.5% Zn	25 days (45°C, pH 1)	Ghassa et al., 2014
Galena concentrate: PbS (38.26 wt.%) ZnS (5.22 wt.%) CuFeS_2_ (5.55 wt.%)	LB medium supplemented with 5 g/L FeCl_3_, 50 g/L NaCl, 20 g/L molasses (2–5% w/v pulp density)	An iron- and sulfur-oxidizing mixotrophic bacterium (*Citrobacter* sp.)	90 g Pb/kg concentrate (=1.8 g/L Pb) 2.3 g Zn/kg concentrate (=46 mg/L) 1.9 g Cu/kg concentrate (=39 mg/L Cu)	7 days (25°C, pH 3–4.7)	Current study

Therefore, the specific aims of the current study were: (1) to investigate biohydrometallurgical leaching process of Indonesian galena concentrates (the main composition of PbS, CuFeS_2_, and ZnS) by the local mixotrophic bacterium *Citrobacter* sp. at room temperature (25°C) by employing two different bioleaching methods (i.e., semi-direct and direct bioleaching) and utilizing the LB medium supplemented with FeCl_3_, NaCl and molasses to minimize the formation of PbSO_4_ precipitates, which had the low solubility of lead within a sulfate system, (2) to evaluate the effect of different molasses concentrations on lead bioleaching efficiency since the bacterium *Citrobacter* sp. produced high amounts of biosurfactants by consuming organic carbon (herein molasses), and (3) to assess the effect of different NaCl and FeCl_3_ concentrations on lead bioleaching efficiency since the presence of NaCl and FeCl_3_ enhanced lead recovery. The findings of this study may provide further insights into the bioleaching of galena that is rarely studied due to insoluble anglesite formation and the toxicity of high lead content in galena to microbes. For our knowledge, this is the first report on the selective bioleaching of lead sulfide ores (herein galena concentrate) from Indonesia that is always associated with sphalerite and chalcopyrite using the local mixotrophic bacterium *Citrobacter* sp. in molasses-supplemented medium containing high NaCl concentration, which thus has potential industrial application since most of the metallurgy and mining industries use seawater in their mineral processing operations.

## Materials and Methods

### Bacterium and Growth Medium

A local mixotrophic bacterium used in this study was isolated from an Indonesian mine site (designated SKC2), which has the ability to oxidize iron and sulfur and produce extracellular polymeric substances (EPS) (Mubarok et al., 2017). Based on the 16S rRNA sequencing analysis, this bacterium was identified as *Citrobacter* sp. (98.45% similarity). The LB broth medium (10 g/L tryptone, 5 g/L yeast extract, 10 g/L NaCl) was used for both semi-direct and direct bioleaching experiments since the preliminary experiments in screening the appropriate medium for lead bioleaching with this bacterium showed the higher lead extraction than the modified Fe-broth medium [0.5 g/L MgSO_4_.7H_2_O, 3 g/L (NH_4_)_2_SO_4_, 0.5 g/L K_2_HPO_4_, 0.1 g/L KCl, 0.5 g/L tryptone, 5 g/L Na_2_S_2_O_3_.5H_2_O, and 1 g/L FeSO_4_.7H_2_O]. In addition, molasses was obtained from a sugar company, Padalarang, West Java, Indonesia.

### Galena Concentrate

The galena concentrate employed in this study was kindly provided by an Indonesian mining company in Bogor, West Java, Indonesia (06°29′ S and 106°33′ E), with a particle size of *d*_80_ = 75 μm. ED-XRF analysis of the concentrate determined its chemical composition, as summarized in Table 3. X-ray powder diffractometry (XRD) analysis showed its mineralogical composition in which galena was predominant in the concentrate sample with a low amount of other minerals such as chalcopyrite and sphalerite (data not shown).

**TABLE 3:** Elemental composition of Indonesian galena concentrate used in this study.

Element	wt. (%)
Pb	38.26
Fe	8.73
S	8.00
Cu	5.55
Zn	5.22
Si	1.04
Al	0.365
Mg	0.157
Mn	0.0826
Ca	0.0623
Cd	0.0540
K	0.0496
As	0.0370
U	0.0208
Ga	0.0186
Se	0.0132
P	0.0116
Ti	0.0056

### Experimental Procedure

Two leaching experiments were performed in this study: (1) semi-direct bioleaching experiments, and (2) direct bioleaching experiments. Bioleaching experiments were conducted in duplicate in sterile 300 ml Erlenmeyer flasks containing 150 ml of bacterial growth medium (LB medium) under aerobic conditions. The growth medium was then supplemented with various concentrations of FeCl_3_ (5, 15, and 25 g/L FeCl_3_), NaCl (30 and 50 g/L NaCl) and molasses (10, 20, and 30 g/L molasses) at various pulp densities (2 and 5% *w/v*) of the galena concentrate, and the pH was adjusted to 4.0 with HCl. Molasses was supplemented to enable the bacterium *Citrobacter* sp. to generate large amounts of biosurfactants (including EPS), thus being capable of preventing ferric (Fe^3+^) ions and PbSO_4_ precipitation. Bacterial inoculum (15% *v/v*) was subsequently introduced into bioleaching suspension, and the cultures were then incubated for 7 days at room temperature (25°C) with shaking at 180 rpm. Periodically, the pH of the suspension was measured using a pH meter (Lutron PE-03), while the redox potential (Eh) of the suspension was measured using an ORP electrode with Ag/AgCl reference (Lutron ORP-14). The solution (5 ml) was removed daily for measuring dissolved metal concentration by using atomic absorption spectrophotometer (AAS; Shimadzu AA-6300, Japan). The percentage of metal extraction (Pb, Cu, and Zn) and selectivity for lead leaching by iron- and sulfur-oxidizing mixotrophic bacterium to Cu (S_Cu_) and to Zn (S_Zn_) were calculated using the equations as described in our earlier work (Chaerun et al., 2017). After 7 days of bioleaching, separate sets of samples (i.e., the galena concentrate residues resulting from the experiments which led to the highest lead extraction for both semi-direct and direct bioleaching experiments) were made up and prepared for analysis by X-ray powder diffraction analysis (XRD; Rigaku Smartlab), Fourier transform infrared (FTIR Prestige 21, Shimadzu, Japan) and scanning electron microscopy equipped with energy dispersive spectroscopy (SEM-EDS; JEOL JSM-J6510 A). Samples were washed three times with deionized water before being observed by FTIR and XRD, while XRD measurement was carried out using Cu-*K*α radiation, generated at 40 kV and 30 mA, using the 2θ/θ method at a scan speed of 2^o^/min. For SEM-EDS observation, samples were fixed with 2.5% *v/v* glutaraldehyde in 5 mM phosphate buffer at pH 7.0 for 24 h at 4°C, washed twice with 5 mM phosphate buffer, dehydrated in a graded series of acetone (25, 50, 75, and 100%) for 15 min, 15 min, 15 min, and 24 h, respectively.

For semi-direct bioleaching experiments, the galena concentrate was introduced into the culture medium after 3 days of incubation. For direct bioleaching experiments, the concentrate was introduced into the medium at the onset of the experiments. In comparison, the abiotic control leaching experiments were also conducted to ensure the bacterial participation in lead bioleaching processes, which were identical to those for semi-direct and direct bioleaching experiments, except that the bacterium was not added. The data are presented as the averages obtained from the duplicate experiments, and error bars represent standard deviation. Also, biosurfactant production by the mixotrophic bacterium during its bacterial growth for 48 h was assayed by measuring the emulsifying activity index (EI, %) following the work of Berg et al. (1990) with modification (Chaerun et al., 2018). This assay was conducted to confirm the role of the generated biosurfactants (including EPS as high-molecular-weight biosurfactants) in promoting lead bioleaching efficiencies. Briefly, the sample (2 mL) of the bacterial culture broth was mixed with 5 mL of TM buffer, which contained 20 mmol Tris-HCl buffer (pH 7.0) and 10 mmol MgSO_4_.7H_2_O per liter of deionized water, followed by addition of 1 mL coconut oil. After the mixture was vortexed for 3 min and incubated at room temperature for 1 min, the first turbidity (A1) of the aqueous phase was measured at 600 nm. Subsequently, after the mixture was incubated at room temperature for 60–90 min, the second turbidity (A2) of the aqueous phase was measured. Emulsifying activity index (EI, %) was then expressed in Eq. (1).

(1) $$E\,I=\left(1-\frac{O\,D_{600}\,\text{of}\,\text{A}\,2}{O\,D_{600}\,\text{of}\,\text{A}\,1}\right)\times 100$$

## Results and Discussion

### Semi-Direct Bioleaching

**FIGURE 1:**
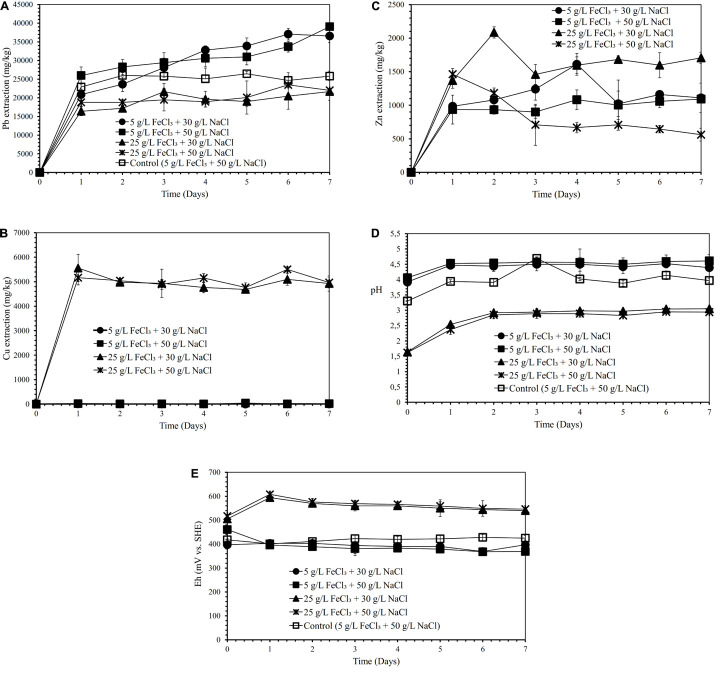
Lead (Pb) extraction (mg Pb dissolved/kg galena concentrate) **(A)**, Copper (Cu) extraction (mg Cu dissolved/kg galena concentrate) **(B)**, Zinc (Zn) extraction **(C)** (mg Zn dissolved/kg galena concentrate), pH **(D)** and Eh (mV vs. SHE) **(E)** of the galena concentrate semi-direct bioleaching suspension by an iron- and sulfur-oxidizing mixotrophic bacterium (*Citrobacter* sp.) at various concentrations of FeCl_3_ (5 and 25 g/L) and NaCl (30 and 50 g/L) at bioleaching parameters of 15% *v/v* bacterial inoculum, 5% *w/v* pulp density, 10 g/L molasses, a rotation speed of 180 rpm over a 7-day period of the experiment in comparison with those of the abiotic control semi-direct leaching (without bacteria) at 5 g/L FeCl_3_ and 50 g/L NaCl.

Semi-direct bioleaching experiments were conducted by adding various concentrations of FeCl_3_ (5 and 25 g/L), NaCl (30 and 50 g/L) and molasses (10, 20, and 30 g/L) at 5% *w/v* pulp density to evaluate their effects on lead recovery. Figures 1A–C demonstrate the effect of various concentrations of FeCl_3_ (5 and 25 g/L) and NaCl (30 and 50 g/L) on lead, copper and zinc extraction (mg metals of Pb, Cu, Zn dissolved per kg concentrate; mg/kg) by iron-and sulfur-oxidizing mixotrophic bacterium (*Citrobacter* sp.) in the semi-direct bioleaching of the galena concentrate over a 7-day period of the experiment at bioleaching parameters of 15% *v/v* bacterial inoculum, 5% *w/v* pulp density, and 10 g/L molasses. Lead bioleaching efficiencies from the concentrate increased rapidly during the first 1 day and subsequently increased slightly for the remaining bioleaching time, which achieved the extraction levels of 37–39 g/kg (for the addition of 5 g/L FeCl_3_ with 30 g/L or 50 g/L NaCl) and 22 g/kg (for the addition of 25 g/L FeCl_3_ with 30 g/L or 50 g/L NaCl) (Figure 1A). These increments were concomitant with an increase in copper and zinc bioleaching efficiencies (Figures 1B,C), except for copper dissolution with the addition of 5 g/L FeCl_3_ with 30 g/L or 50 g/L NaCl which was negligible (Figure 1B). Zinc dissolution was quite low compared to lead and copper extraction, thus being considered to be selective bioleaching to zinc. No difference was observed for lead extraction from the galena concentrate at any NaCl concentration (30 g/L or 50 g/L NaCl), and its extraction was apparently governed by the presence of FeCl_3_, of which 5 g/L was the best concentration (Figure 1A). The presence of FeCl_3_ also affected the initial pH of the suspension, where the higher FeCl_3_ concentration introduced into the solution yielded the more acidic suspension (Figure 1D). This lower pH was a result of the hydrolysis of FeCl_3_, generating HCl, which therefore lowered the suspension pH. Moreover, the pH of the suspensions tended to increase over time as a result of sulfide mineral oxidation that consumed proton (H^+^), whereas the suspension Eh values containing the higher FeCl_3_ concentration (25 g/L) were observed to be higher than those containing the lower FeCl_3_ concentration (5 g/L) due to the oxidizing capacity of FeCl_3_, which had a more oxidizing agent of Fe^3+^ (Figure 1E). Since the semi-direct bioleaching at 5 g/L FeCl_3_ and 50 g/L NaCl was highly selective to Cu (*S*_Cu_ = 0.99) and was relatively selective to Zn (*S*_Zn_ = 0.83) (Figures 1B,C and Table 4), hereafter the concentration was used for further bioleaching experiments to enhance the extraction of Pb as well as to prevent the formation of PbSO_4_ precipitates as well as ferric (Fe^3+^) ion precipitation by adding organic compounds (herein molasses).

**TABLE 4:** The selectivity of lead (Pb) bioleaching by an iron- and sulfur-oxidizing mixotrophic bacterium (*Citrobacter* sp.) to Cu (S_Cu_) and Zn (S_Zn_) based on the effect of varying concentrations of FeCl_3_, NaCl and molasses as well as different bioleaching methods (Figures 1–3) over a 7-day period of bioleaching experiment.

Time (Day)	Semi-direct bioleaching (addition of FeCl_3_ + NaCl)								Semi-direct bioleaching (addition of molasses)						Direct bioleaching (addition of FeCl_3_)			
	5 + 30 (g/L)		5 + 50 (g/L)		25 + 30 (g/L)		25 + 30 (g/L)		10 (g/L)		20 (g/L)		30 (g/L)		5 (g/L)		15 (g/L)	
	*S*_Cu_	*S*_Zn_	*S*_Cu_	*S*_Zn_	*S*_Cu_	*S*_Zn_	*S*_Cu_	*S*_Zn_	*S*_Cu_	*S*_Zn_	*S*_Cu_	*S*_Zn_	*S*_Cu_	*S*_Zn_	*S*_Cu_	*S*_Zn_	*S*_Cu_	*S*_Zn_
1	0.99	0.74	0.99	0.79	0.30	0.62	0.35	0.64	0.99	0.79	0.99	0.80	0.99	0.80	0.78	0.72	0.58	0.75
2	0.99	0.75	0.99	0.81	0.33	0.53	0.35	0.68	0.99	0.81	0.99	0.80	0.99	0.83	0.80	0.74	0.54	0.67
3	0.99	0.76	0.99	0.82	0.39	0.67	0.37	0.79	0.99	0.82	0.99	0.83	0.99	0.83	0.81	0.77	0.54	0.70
4	0.99	0.74	0.99	0.79	0.37	0.63	0.35	0.80	0.99	0.79	0.99	0.82	0.99	0.83	0.80	0.76	0.52	0.71
5	0.99	0.82	0.99	0.81	0.37	0.61	0.38	0.80	0.99	0.81	0.99	0.84	0.99	0.82	0.81	0.79	0.50	0.67
6	0.99	0.81	0.99	0.81	0.37	0.64	0.38	0.83	0.99	0.81	0.99	0.86	0.99	0.85	0.84	0.78	0.49	0.69
7	0.99	0.82	0.99	0.83	0.39	0.63	0.39	0.84	0.99	0.83	0.99	0.86	0.99	0.84	0.87	0.84	0.58	0.77

**FIGURE 2:**
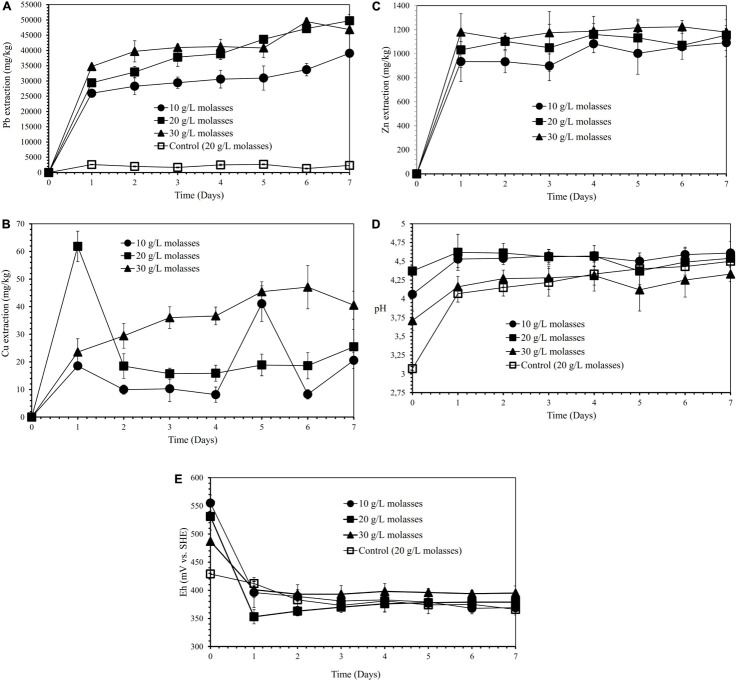
Lead (Pb) extraction (mg Pb dissolved/kg galena concentrate) **(A)**, Copper (Cu) extraction (mg Cu dissolved/kg galena concentrate) **(B)**, Zinc (Zn) extraction **(C)** (mg Zn dissolved/kg galena concentrate), pH **(D)** and Eh (mV vs. SHE) **(E)** of the galena concentrate semi-direct bioleaching suspension by an iron- and sulfur-oxidizing mixotrophic bacterium (*Citrobacter* sp.) at various molasses concentrations (10, 20, and 30 g/L) at bioleaching parameters of 15% *v/v* bacterial inoculum, 5% *w/v* pulp density, 5 g/L FeCl_3_, 50 g/L NaCl, a rotation speed of 180 rpm over a 7-day period of the experiment in comparison with those of the abiotic control semi-direct leaching (without bacteria) at 20 g/L molasses.

Figures 2A–C show the effects of various molasses concentrations (10, 20, and 30 g/L) on lead, copper, and zinc extraction (mg metals of Pb, Cu, Zn dissolved per kg concentrate; mg/kg) by iron- and sulfur-oxidizing mixotrophic bacterium (*Citrobacter* sp.) in semi-direct bioleaching of the galena concentrate over 7 days of the experiment at bioleaching parameters of 15% v/v bacterial inoculum, 5% *w/v* pulp density, 5 g/L FeCl_3_ and 50 g/L NaCl. Lead extraction (39∼50 g/kg) from the concentrate was observed for three different molasses concentrations tested, and molasses concentration of 20 and 30 g/L appeared to bring about higher lead extraction than that of 10 g/L. The lead bioleaching efficiencies from the concentrate increased rapidly during the first 1 day and subsequently increased slightly for another 6 days (Figure 2A). It appeared that the addition of molasses created selective bioleaching, the high selectivity for copper, and the relatively high selectivity for zinc (Figures 2B,C and Table 4). During the bioleaching experiments, the suspension pH tended to increase, while the Eh otherwise decreased (Figures 2D,E). Since 20 g/L molasses was the best concentration for lead extraction with the high selectivity of lead bioleaching to Cu (*S*_Cu_ = 0.99) and Zn (*S*_Zn_ = 0.86), hereafter, the concentration was used in the subsequent experiments (direct bioleaching experiments).

### Direct Bioleaching

**FIGURE 3:**
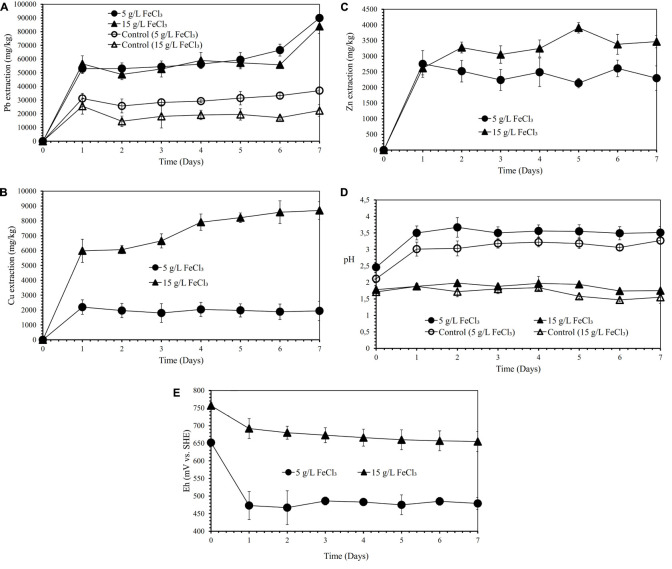
Lead (Pb) extraction (mg Pb dissolved/kg galena concentrate) **(A)**, Copper (Cu) extraction (mg Cu dissolved/kg galena concentrate) **(B)**, Zinc (Zn) extraction **(C)** (mg Zn dissolved/kg galena concentrate), pH **(D)** and Eh (mV vs. SHE) **(E)** of the galena concentrate direct bioleaching suspension by an iron- and sulfur-oxidizing mixotrophic bacterium (*Citrobacter* sp.) and the abiotic control direct leaching (without bacteria) at various FeCl_3_ concentrations (5 and 15 g/L) at bioleaching parameters of 15% *v/v* bacterial inoculum, 2% *w/v* pulp density, 20 g/L molasses, 50 g/L NaCl, a rotation speed of 180 rpm over a 7-day period of the experiment.

Figures 3A–C show the effects of various FeCl_3_ concentrations (5 and 15 g/L) on lead, copper, and zinc extraction (mg metals of Pb, Cu, Zn dissolved/kg galena concentrate) by iron- and sulfur-oxidizing mixotrophic bacterium (*Citrobacter* sp.) in the direct bioleaching of the galena concentrate over a 7-day period of the experiment at bioleaching parameters of 15% *v/v* bacterial inoculum, 2% *w/v* pulp density, 20 g/L molasses, and 50 g/L NaCl. Through the direct bioleaching method, a higher level of Pb extraction (84∼90 g/kg) than semi-direct bioleaching was observed for two FeCl_3_ concentrations (5 and 15 g/L) examined (Figure 3A). However, no significant difference was observed for lead extraction in both FeCl_3_ concentrations. Again, the lead bioleaching efficiencies from galena concentrate increased rapidly during the first 1 day, subsequently remained relatively constant for another 5 days, and slightly increased up to 7 d of bioleaching period. These increases occurred simultaneously with an increase in copper and zinc extraction (Figures 3B,C) with the exception of copper dissolution at 5 g/L FeCl_3_, which was quite low (Figure 3B). It was suggested from the results of this study that the direct bioleaching of galena concentrate at 5 g/L FeCl_3_ led to the high selectivity of lead bioleaching to copper (*S*_Cu_ = ∼0.9) and was relatively selective to zinc (*S*_Zn_ = 0.84) (Figures 3B,C and Table 4). Again, the initial pH of the suspensions containing the higher FeCl_3_ concentration resulted in more acidic suspension, and the pH values of the bacterial suspensions tended to elevate due to sulfide oxidation (Figure 3D). In contrast, the suspension Eh values tended to decline over time (Figure 3E). From the results of the lead extraction (Figures 1A, 2A, 3A), the lead extraction levels in control leaching were lower than those achieved in the bioleaching, indicating that the bacteria greatly participated in the lead bioleaching.

### Characterization of Galena Concentrate Residues After Bioleaching

**FIGURE 4:**
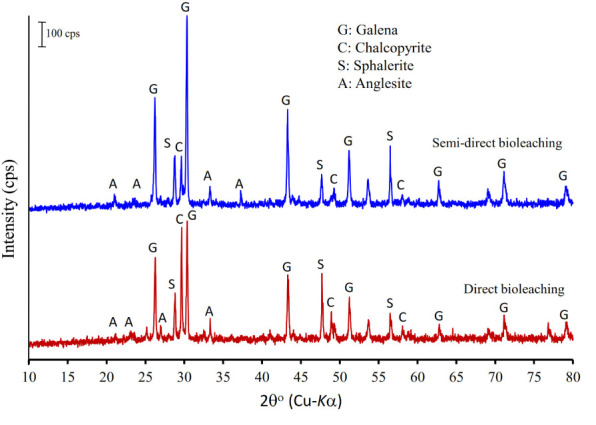
X-ray powder diffraction patterns of the galena concentrate residues in semi-direct bioleaching at bioleaching parameters of 5% *w/v* pulp density, 5 g/L FeCl_3_, 50 g/L NaCl, 20 g/L molasses and in the direct bioleaching at bioleaching parameters of 2% *w/v* pulp density, 5 g/L FeCl_3_, 50 g/L NaCl, 20 g/L molasses by iron- and sulfur-oxidizing mixotrophic bacterium (*Citrobacter* sp.) after 7 days of the bioleaching experiment.

**FIGURE 5:**
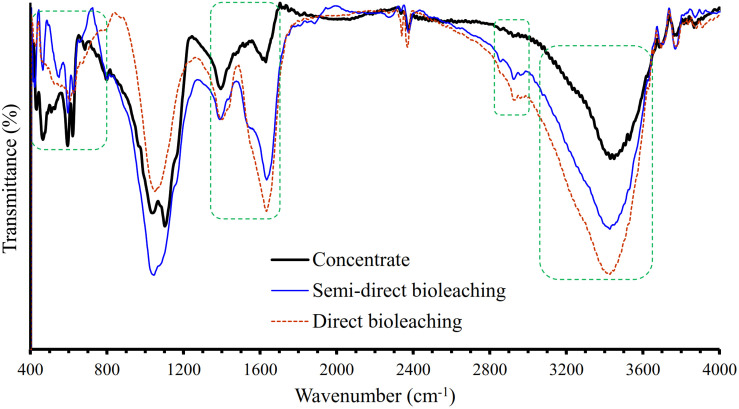
Fourier transform infrared (FTIR) spectra of the galena concentrate residues in semi-direct bioleaching at bioleaching parameters of 5% *w/v* pulp density, 5 g/L FeCl_3_, 50 g/L NaCl, 20 g/L molasses and in the direct bioleaching at bioleaching parameters of 2% *w/v* pulp density, 5 g/L FeCl_3_, 50 g/L NaCl, 20 g/L molasses by iron- and sulfur-oxidizing mixotrophic bacterium (*Citrobacter* sp.) after 7 days of the bioleaching experiment.

Figure 4 shows the XRD patterns of the galena concentrate and its residues in semi-direct bioleaching and direct bioleaching after 7 days of the bioleaching experiments. Both bioleaching techniques led to the formation of anglesite, thus hindering the enhancement of Pb extraction from the concentrate (Figures 2A, 3A). In addition, galena remained as a predominant mineral in leach residues in association with a small amount of chalcopyrite and sphalerite. From the XRD spectra, changes in mineralogical structure due to bioleaching were not discernible; hence FTIR analysis was performed to investigate chemical bondings in galena concentrate before and after bioleaching (Figure 5). The FTIR spectrum of starting galena concentrate contains bands at 400–800 cm^–1^ (inorganic components such as minerals and clays), 621 cm^–1^ (C-S stretching), 1,000–1,230 cm^–1^ (Si-O stretching), ∼1,081 cm^–1^ (C-O stretching vibrations of C-O-C groups, e.g., cellulose), ∼1,400 cm^–1^ (carboxylic and carbonylic groups), 1,620–1,640 cm^–1^ (hydrophilic C=O groups), and 3,000–3,700 cm^–1^ (O-H stretching, H-bonds, and OH-groups). It exhibited that the bands at 400–800 cm^–1^ (inorganic components such as minerals including galena) were reduced after bioleaching (both semi-direct and direct bioleaching processes) but not before the bioleaching process (as galena concentrate) (Figure 5). This reduction could be as a result of the bacterial role in bioleaching lead from galena concentrate, which was also supported by the sharp peaks of the leach residues at 1,385 and 1,535–1,660 cm^–1^ as an obvious indicator of the presence of bacterial cells (Chaerun et al., 2013). Moreover, the increased intensity of a broad band at 3,000–3,600 cm^–1^ (H-bonds and OH-groups) and two peaks at 2,860 and 2,920 cm^–1^ (asymmetric and symmetric C-H stretching vibrations of CH_3_ and CH_2_ groups) were observed for the leach residues. These results indicated that the residues retained more water and hydrophobic organic matters than the concentrates, which corresponded to the presence of extracellular polymeric substances (EPS) generated by the bacteria in this study. This observation is in agreement with the result of Chaerun et al. (2013), who reported that EPS, as a hygroscopic, highly hydrated biopolymer has the ability to retain water entropically. Other works also provide additional support for our FTIR results on EPS generation, demonstrating that the EPS is cell-bound and causes the bacterial cell surface to become more hydrophobic due to the hydrophobic properties of the EPS (Govender and Gericke, 2011) that are attributed to compounds, such as polysaccharide-linked methyl and acetyl groups (Flemming and Wingender, 2010).

**FIGURE 6:**
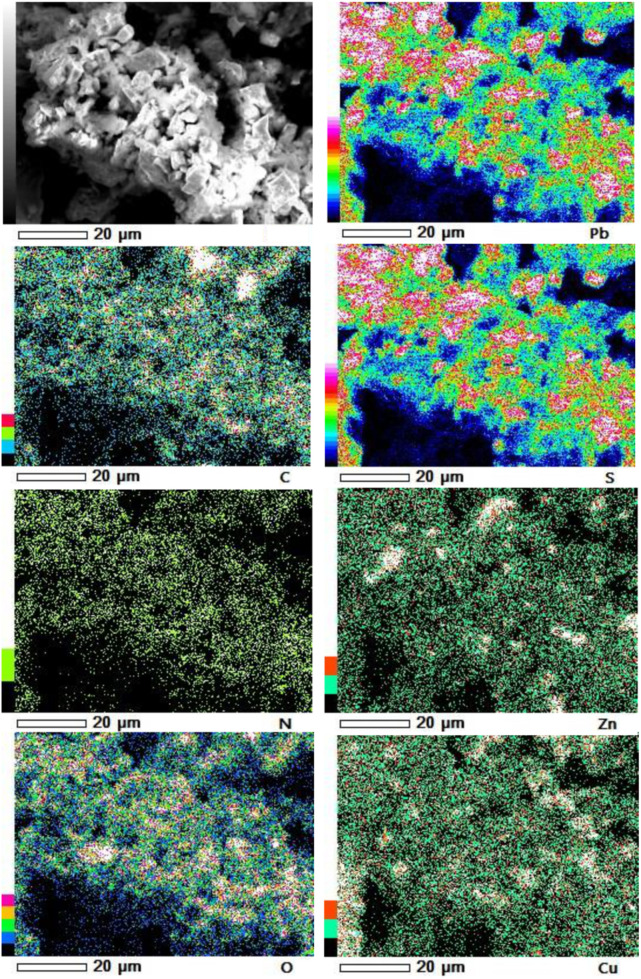
SEM-EDS maps of elemental compositions of the galena concentrate residues in semi-direct bioleaching at bioleaching parameters of 5% *w/v* pulp density, 5 g/L FeCl_3_, 50 g/L NaCl, 20 g/L molasses by iron- and sulfur-oxidizing mixotrophic bacterium (*Citrobacter* sp.) after 7 days of the bioleaching experiment.

**FIGURE 7:**
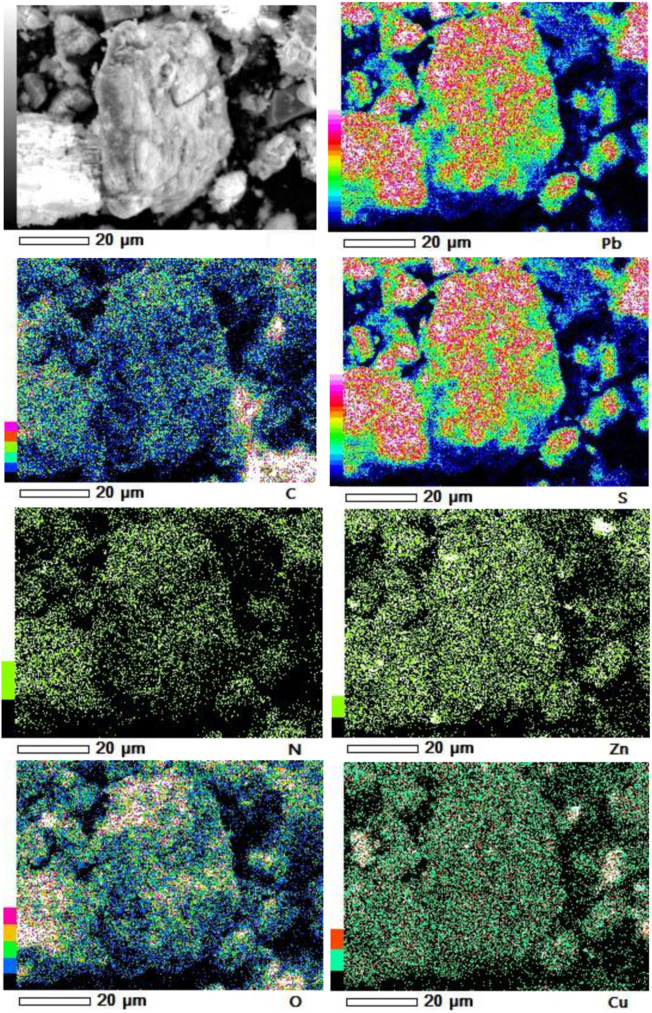
SEM-EDS maps of elemental compositions of the galena concentrate residues in the direct bioleaching at bioleaching parameters of 2% *w/v* pulp density, 5 g/L FeCl_3_, 50 g/L NaCl, 20 g/L molasses by iron- and sulfur-oxidizing mixotrophic bacterium (*Citrobacter* sp.) after 7 days of the bioleaching experiment.

**FIGURE 8:**
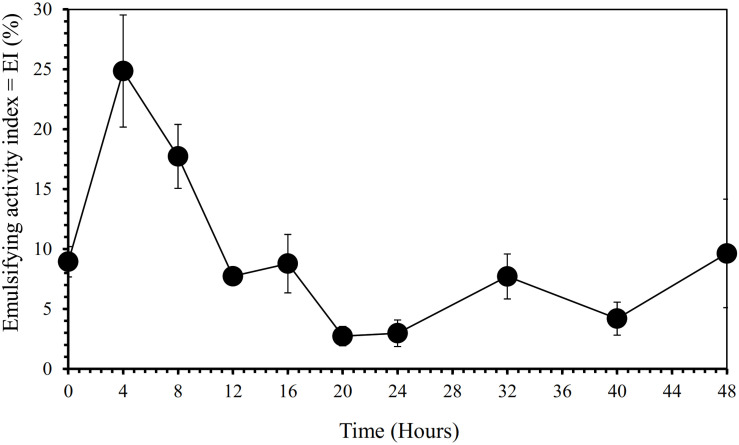
Biosurfactant production by iron- and sulfur-oxidizing mixotrophic bacterium (*Citrobacter* sp.) as represented by emulsifying activity index (EI in %) over 48 h of the bacterial growth in the modified LB medium.

Furthermore, our SEM-EDS mapping observation of the galena concentrate residues (Figures 6, 7) also supports this hypothesis in that an iron- and sulfur-oxidizing mixotrophic bacterium (*Citrobacter* sp.) used in this study generates EPS, which thus forms the EPS-concentrate complexes and is consequently able to promote the interfacial degradation of the galena concentrate as well as the bioleaching of lead from the concentrate. This was represented by the formation of aggregates as a result of bacterial attachment to galena surfaces (due to EPS), which was also evidenced by the presence of C and N in the residues as the main component of bacterial cells and EPS content (Figures 6, 7). Small amounts of Cu and Zn revealed by SEM-EDS mapping observation in this study also confirmed the bioleaching of lead, copper, and zinc, as shown in Figures 2, 3. This result was also supported by the elemental contents of the residues (Table 5), demonstrating larger amounts of C, N, P, and smaller amounts of Pb, Cu, Zn in the residues than in the concentrate. In addition to FTIR and SEM-EDS observations, the role of EPS generated by the bacterium in elevating the lead bioleaching efficiencies rapidly during the first 1 day (Figures 1A, 2A, 3A) was also confirmed by the biosurfactant production (as represented by emulsifying activity index = EI) of the bacterium over 48 h of bacterial growth in the modified LB medium, demonstrating that the highest biosurfactant production was attained at 4 h of bacterial growth (Figure 8).

**TABLE 5:** Elemental concentration (**^a^**) of Indonesian galena concentrate after 7 days of bioleaching by an iron- and sulfur-oxidizing mixotrophic bacterium (*Citrobacter* sp.).

Element	wt. (%)
Pb	7.18 (0.19) ∼ 18.63 (0.09)^b^
Fe	3.15 (1.28)
S	11.99 (2.58)
Cu	1.34 (0.56)
Zn	n.d.
Si	0.03 (0) ∼ 0.47 (0)
Al	0.28 (0.005)
Mg	0.04 (0.01)
Mn	n.d.
Ca	0.03 (0)
Cd	0.07 (0)
K	–
As	2.14 (0.43)
U	n.d.
Ga	n.d.
Se	n.d.
P	0.38 (0.02)
Ti	0.04 (0)
C	40.7 (1.3)
N	6.4 (0.5)
O	20.36 (3.28)

**n.d., not detected; “–”, not measured; ^*a*^based on quantitative ED-XRF analyses (n = 2–4); ^*b*^standard deviation*.*

From the results of this study, it could be seen that both semi-direct and direct bioleaching took place at the pH range of 1.6∼4.6 at which the bacterium *Citrobacter* sp. used in this study was able to proliferate, which had pH optima near neutrality and minimum and maximum pH values for growth 1.5 and 9.5, respectively. Meanwhile, both semi-direct and direct bioleaching exhibited the elevated lead extraction levels (∼50 and ∼90 g/kg, respectively) compared with copper (∼0.03 and ∼2 g/kg, respectively) and zinc (∼1 and ∼2.3 g/kg, respectively) extraction levels (Figures 2A–C, 3A–C), which were attained at the addition of 5 g/L FeCl_3_, 50 g/L NaCl, 20 g/L molasses at pulp density of 5% *w/v* (for semi-direct bioleaching) and 2% *w/v* (for direct bioleaching). The mixotrophic bacterium *Citrobacter* sp. (as a single culture) which exhibited the excellent selectivity of lead bioleaching to copper and zinc might be more beneficial for subsequent copper and zinc leaching processes than chemolithoautotrophic bacteria used in previous studies (listed in Table 2), which were not selective to zinc (Pacholewska, 2004; Baba et al., 2011; Ghassa et al., 2014), while selectivity for copper was not determined (Jiang et al., 2008; Mejía et al., 2012). It is reported that galena (PbS) that is often associated with zinc sulfides such as sphalerite (ZnS) and copper sulfides such as chalcopyrite (CuFeS_2_) frequently makes passivation layers that hinder Cu and Zn dissolution (Dutrizac, 1986; Dutrizac and Chen, 1990; Da Silva et al., 2003).

The overall reaction of PbS dissolution in the presence of FeCl_3_ and NaCl and the Gibbs free energy for PbS oxidation are presented in Eqs. (2)–(8) (Shock et al., 1997; Chase, 1998). According to the Gibbs free energy values, the reactions had negative values, indicating the spontaneous reaction. However, the negative values obtained said nothing about the kinetics because the kinetics was affected by the slowest reaction step in the leaching process. For example, Gibbs free energy for the dissolution of chalcopyrite (CuFeS_2_) in acidified ferric sulfate solution was negative (Hiroyoshi et al., 2000), but in fact, the copper extraction level in this system was very low, and long periods of time and high temperature were needed for the complete dissolution (Dutrizac, 1978). Moreover, if the dissolution process brings about non-porous solid products on the mineral surfaces, then the products can preclude the leaching agents such as ferric iron from reacting with the minerals and thus retard the kinetics, while the reactions might proceed faster or slower. The presence of bacteria acts as the catalyst, thus accelerating the reactions. In this study, the bacterium *Citrobacter* sp. was shown to increase Pb dissolution from the galena concentrate compared to abiotic control leaching without bacteria.

The oxidation reaction of PbS by ferric ions:

(2) $$\begin{array}{l} PbS_{\left(s\right)}+2Fe_{\left(aq\right)}^{3+}\to Pb_{\left(aq\right)}^{2+}+2Fe_{\left(aq\right)}^{2+}+S_{\left(s\right)}^{0}\\ \;{\text{ΔG}}_{r1,298\text{K}}=-76.1kJ/mol \end{array}$$

Oxidation reaction of elemental sulfur and ferrous ions by bacteria:

(3) 2Fe(aq)2++0.5O2(aq)+2H(aq)+→bacteria2Fe(aq)3++H2O(l)                                                                   ΔG=r2,298K−88.6kJ/mol

(4) S(s)0+0.5O2(aq)+H2O(l)→bacteriaH2SO4(aq)                                                          ΔG=r3,298K−452.8kJ/mol

Reaction of Pb ions with chloride or sulfate ions:

(5) Pb(aq)2++Cl(aq)−→PbCl(aq)+                                          ΔG=r4,298K−7.9kJ/mol

(6) Pb(aq)2++2Cl(aq)−→PbCl2(aq)                                            ΔG=r5,298K−11.1kJ/mol

(7) $$\begin{array}{l} Pb_{\left(aq\right)}^{2+}+3Cl_{\left(aq\right)}^{-}\to PbCl_{3(aq)}^{-}\\ {\text{ ΔG}}_{\text{r}6,298\text{K}}=-9.4kJ/mol \end{array}$$

(8) $$\begin{array}{l} Pb_{\left(aq\right)}^{2+}+SO_{4(aq)}^{2-}\to PbSO_{4(s)}\\ \;{\text{ΔG}}_{r7,298\text{K}}=-44.3kJ/mol \end{array}$$

Correspondingly, the molasses introduced into the bioleaching systems enabled the mixotrophic bacterium *Citrobacter* sp. to gain the energy needed to carry out its metabolic processes from organic compounds (as organic carbon source) in the presence of oxygen in which the bacterium was essential participants in the complete oxidation of organic compounds (CH_2_O) to CO_2_ + H_2_O along with a much greater yield of energy [Eq. (9)]. By utilizing the organic carbon, the bacterium was capable of producing large amounts of biosurfactants (including EPS), which mediated tight bacterial adherence to galena concentrate as well as kept solubilizing ferric iron even at pH > 4.0 as a result of complexation of the ferric ion with EPS (Vardanyan et al., 2020).

(9) $$\begin{array}{l} CH_{2}O+O_{2(g)}\to CO_{2(g)}+H_{2}O_{\left(l\right)}\\ \;{\text{ΔG}}_{r8,298\text{K}}=-500.4kJ/mol \end{array}$$

In summary, the current study presents a biohydrometallurgical leaching process of the Indonesian galena concentrate containing chalcopyrite and sphalerite at room temperature and atmospheric pressure, which exhibits selective leaching of lead to copper and zinc where this leaching technique prevents the release of SO_2_ and lead (Pb) dust emission. It is necessary to conduct further research to obtain the optimum lead bioleaching conditions by adapting the bacterium to galena concentrate first before employing it in the bioleaching systems since the current leaching study uses non-adapted bacteria with a low pulp density (2% *w/v*). By having the adapted bacteria, the higher pulp density in the leaching process can be increased, and Pb toxicity of higher pulp density can be reduced. Compared to the smelting technology currently used in the Pb extraction from galena (PbS), the biohydrometallurgical Pb leaching can potentially compete because of its advantage in the following aspects: more eco-friendly method, energy-saving leaching process, selective leaching of Pb to Cu and Zn. The selective Pb leaching to Cu and Zn demonstrated in this study thus makes a subsequent Cu and Zn extraction much easier in the downstream process (through biohydrometallurgical or hydrometallurgical route) since Pb has been removed. Moreover, the bacterium *Citrobacter* sp. in this study has shown to have the capacity to extract Pb from galena concentrate in the LB medium containing high NaCl concentration, therefore in the further research, the use of NaCl can be replaced with seawater, which is commonly used in mining and metallurgy industries, whereas tryptone can be substituted by molasses.

## Conclusion

The present study has shown that an iron- and sulfur-oxidizing mixotrophic bacterium (*Citrobacter* sp.) used in this study is capable of extracting lead (Pb) from the Indonesian galena concentrate which achieves the highest extraction level of 90 g lead dissolved/kg galena concentrate using direct bioleaching method at bioleaching parameters of 2% *w/v* pulp density, 5 g/L FeCl_3_, 50 g/L NaCl, 20 g/L molasses and a rotation speed of 180 rpm at room temperature (25°C). The results of this study may, therefore, be advantageous to the improvement of Pb leaching from sulfide ores through a more environmentally friendly biohydrometallurgical route.

## Data Availability Statement

The raw data supporting the conclusions of this article will be made available by the authors, without undue reservation, to any qualified researcher.

## Author Contributions

EAP performed the experiments under the supervision of SKC. SKC wrote the manuscript, while MZM made figures and tables. All authors revised the manuscript.

## Conflict of Interest

The authors declare that the research was conducted in the absence of any commercial or financial relationships that could be construed as a potential conflict of interest.
